# Socio-cognitive profiles for visual learning in young and older adults

**DOI:** 10.3389/fnagi.2015.00105

**Published:** 2015-06-10

**Authors:** Julie Christian, Aimee Goldstone, Shu-Guang Kuai, Wynne Chin, Dominic Abrams, Zoe Kourtzi

**Affiliations:** ^1^School of Psychology, University of BirminghamBirmingham, UK; ^2^Key Laboratory of Brain Functional Genomics, Ministry of Education, Shanghai Key Laboratory of Brain Functional Genomics, East China Normal UniversityShanghai, China; ^3^Department of Decision and Information Sciences, Bauer College of Business, University of HoustonHouston, TX, USA; ^4^Department of Psychology, University of KentCanterbury, UK; ^5^Department of Psychology, University of CambridgeCambridge, UK

**Keywords:** perceptual learning, visual perception, psychophysics, cognitive abilities, social profiles, individual differences

## Abstract

It is common wisdom that practice makes perfect; but why do some adults learn better than others? Here, we investigate individuals’ cognitive and social profiles to test which variables account for variability in learning ability across the lifespan. In particular, we focused on visual learning using tasks that test the ability to inhibit distractors and select task-relevant features. We tested the ability of young and older adults to improve through training in the discrimination of visual global forms embedded in a cluttered background. Further, we used a battery of cognitive tasks and psycho-social measures to examine which of these variables predict training-induced improvement in perceptual tasks and may account for individual variability in learning ability. Using partial least squares regression modeling, we show that visual learning is influenced by cognitive (i.e., cognitive inhibition, attention) and social (strategic and deep learning) factors rather than an individual’s age alone. Further, our results show that independent of age, strong learners rely on cognitive factors such as attention, while weaker learners use more general cognitive strategies. Our findings suggest an important role for higher-cognitive circuits involving executive functions that contribute to our ability to improve in perceptual tasks after training across the lifespan.

## Introduction

Successful interactions in dynamic environments are known to benefit from past experience. But why do some adults learn better than others? Despite the general consensus that practice makes us “perfect”, the striking variability in learning ability among individuals remains largely unexplained (Ackerman, 1987; Saarinen and Levi, 1995; Withagen and Van Wermeskerken, 2009). Previous behavioral, neurophysiology and neuroimaging studies (for reviews, see e.g., Gilbert et al., 2001; Fine and Jacobs, 2002; Kourtzi and DiCarlo, 2006) have advanced our understanding of the learning mechanisms that facilitate behavioral improvements through training; yet the socio-cognitive factors that underlie individual variability in learning ability remain largely unknown.

In this study, we sought to understand the roles of cognitive and social capacities that may underlie individual variability in our ability to improve in perceptual tasks through training (cf. Hutchens et al., 2013). Training is shown to facilitate performance in a wide range of perceptual skills in both young (Fine and Jacobs, 2002; Sagi, 2011) and older adults (Ball and Sekuler, 1986; Richards et al., 2006; Andersen et al., 2010; Bower and Andersen, 2012). For instance, recent studies show that training enhances performance on a wide range of tasks, including brightness discrimination (Ratcliff et al., 2006), acuity (Fahle, 1993), texture discrimination (Andersen et al., 2010), motion direction discrimination (Ball and Sekuler, 1986; Bower and Andersen, 2012; Bower et al., 2013) and global form perception tasks (Kuai and Kourtzi, 2013).

**Figure 1:**
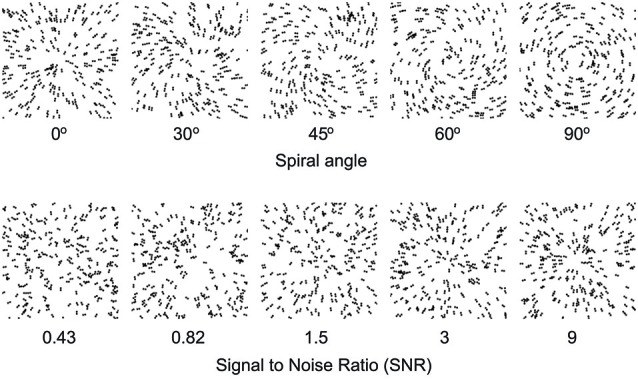
**Example stimuli**. Examples of Glass pattern stimuli (stimulus parameters are adjusted for demonstration purposes). The top panel shows Glass patterns stimuli with different spiral angles from radial (0°) to concentric (90°). The bottom panel shows radial Glass patterns at different levels of signal-to-noise-ratio (SNR) from 0.43 to 9.

To understand how learning improves our ability to recognize objects, we trained young and older participants on a global form discrimination task that entails extracting task-relevant information from distracting background noise similar to identifying a friend in the crowd or a familiar object in a cluttered scene. In particular, we used parametric manipulations of Glass patterns that comprise oriented dot dipoles (Figure 1). For these stimuli, small local changes to dot patterns have a predictable influence on the perception of global forms (concentric vs. radial patterns). Further, adding background noise (i.e., randomly oriented dipoles) increases the difficulty of the task and worsens our ability to discriminate between global patterns. Our previous work (Kuai and Kourtzi, 2013) has shown that training on this task improves global form discrimination in both young and older adults. However, tolerance to external noise varies across individuals, especially in older age, suggesting that visual selection processes may impose limits to perceptual learning and result in individual variability. Thus, we predict that variability in perceptual learning tasks may relate to cognitive (i.e., attentional, memory) skills that facilitate extracting relevant information while suppressing distracting patterns.

To this end, we developed a battery of cognitive tests and theory-grounded individual differences measures, assessing the extent to which cognitive abilities combine with individual strategies to determine learning performance. Next, we sought to relate cognitive and social profiles from participants of all ages to individual learning ability in the context of a visual discrimination task. We asked whether an individual’s cognitive and social skills profile predicts training-induced improvements in perceptual tasks and tested for abilities that mediate learning independent of chronological age. While there is no single guiding theory that explains learning-dependent improvement in nonverbal tasks, previous studies have linked accuracy of learning to variables such as individual’s social perceptions (including cognitive style; Sternberg and Zhang, 2011), motivation (Dweck, 1986; Lau and Roeser, 2008), self-confidence and self-esteem (Lamont et al., 2015). Thus, we tested the hypothesis that these social factors may interact with cognitive processes that support our ability to extract relevant information and facilitate behavioral improvement in perceptual tasks through training in young and older adults.

## Materials and Methods

### Participants

Sixty participants, 30 young adults (11 male and 19 female ranging in age from 19 to 36 years old, *M* = 21.43), and 30 older adults (17 male and 13 female, ranging in age from 65 to 90 years, *M* = 73.60) took part in the study. All participants had normal to corrected vision and underwent the following visual tests: Visual acuity (Bailey-Lovie near and far acuity tests; Bailey and Lovie, 1976), contrast sensitivity (Pelli-Robson Contrast Sensitivity test; Pelli et al., 1988), and color blindness. None of the participants had been exposed previously to the task. Older participants also completed the Mini Mental exam (Folstein et al., 1975), and all scored within the normal range (25–30). This study was approved by the University of Birmingham Ethics Committee.

#### Recruitment

Two strategies were used to guide recruitment. Young participants were recruited from university’s research participation scheme; whereas, older participants were recruited from the university’s database for research into aging (which is drawn from university alumni and therefore indicates a secondary education), or by adverts placed in local publications. Most participants reported having some educational background (16 were University alumni, 6 reported no educational achievement). Comparison of means indicates that there are no significant differences between older participants scores on study variables due to avenue of recruitment. Further, older participants in our study were in frequent contact with research groups in the university and were regularly asked to participate in various studies. Thus, they were likely to have had exposure to the same types of equipment and questionnaires as the younger adults in the study. Importantly, our experiments were conducted by well-trained researchers that explained in detail and repeatedly the tasks and instructions and monitored closely progress in the experiment, ensuring that participants were familiar with the computer equipment and understood well all aspects of the task and instructions in questionnaires. Finally, all young and older participants were paid for their participation.

### Visual Learning Task

We used Glass patterns (Glass, 1969) as stimuli that are generated by pairing randomly positioned dots to form dipoles (dot pairs). Our stimuli comprised 600 white dot pairs (dipoles) displayed within a square aperture (7.5 × 7.5°) on a black background. Each dipole consisted of two 0.0375° dots with 0.26° separation between them. By placing dipoles tangentially and orthogonally to the circumference of a circle centered on the fixation dot we created concentric and radial patterns respectively. We also generated intermediate Glass patterns between these two pattern types by parametrically varying the spiral angle between 0° (radial) to 90° (concentric). The spiral angle was defined as the angle between the dot dipole orientation and the radius from the center of the dipole to the center of the stimulus aperture. Further, we manipulated signal-to-noise ratio (SNR; i.e., the ratio of signal dipoles to noise dipoles: randomly positioned and oriented dipoles) and presented stimuli at 30%, 45%, 60%, 75%, 90%, and 100% signal corresponding to SNR of 0.43, 0.82, 1.5, 3, 9 and ∞, respectively. We set the lowest SNR at the detection threshold of Glass patterns in noise (29.8 ± 1.59% signal) as indicated by our pilot and previous studies (Kuai and Kourtzi, 2013). A new pattern was generated for each stimulus presented in a trial, resulting in stimuli that were locally jittered in their position. These parameters were chosen based on pilot psychophysical studies and in accordance with previous work (Wilson and Wilkinson, 1998; Kuai and Kourtzi, 2013) showing that coherent form patterns are reliably perceived for these parameters. All participants were familiarized with the task and stimuli during a short practice session (100 trials). Participants took part in one pre- and one post-training session without error feedback and four to five training sessions with feedback. Feedback was delivered by an auditory signal (beep) when the participants responded incorrectly in a trial. On each trial, a stimulus pattern was presented for 200 ms followed by a 500 ms blank screen. Participants were instructed to report whether the pattern was radial or concentric. We measured participants’ performance using a 3-down-1-up staircase method resulting in 79.4% convergence level. That is, task difficulty increased following correct response in three trials, while it decreased following one incorrect response. Task difficulty was manipulated by changing the spiral angle (i.e., spiral angles closer to 0° or 90° corresponding to radial and concentric patterns were easier to discriminate than spiral angles closer to 45°). We measured spiral angle thresholds by averaging the last two-third reversals in each staircase. In the pre- and post-training sessions, we measured participants’ performance using three to five staircases with nine or ten up-down reversals at each SNR. In each training session, participants received training on 1200–2000 trials with feedback.

### Individual Differences Measures

Previous research exploring individual variability in aging has typically used measures of control beliefs and self-reported measures of personal approaches as a means for detecting individual variability. However, these measures are more indirect and general in their focus. Here, we develop a more direct measure of the influence of individual differences and motivational factors on learning. In particular, we investigated learning styles (Biggs, 1988; Evans et al., 2009; Sadler-Smith et al., 2009) within the context of the applied social cognition framework drawn from work exploring intrinsic motivation (Dweck, 1986) and the influence of personal self-esteem (Rosenberg et al., 1995). Additionally, evidence from the field of social psychology suggests that *style of thinking* (Sternberg’s Theory of Mental Self Government; Sternberg, 1988, 1997; Sternberg and Grigorenko, 1997) or *learning approach* (J B. Biggs’ “Theory of Learning Approaches”; Biggs, 1988), influences learning outcome (Gully and Chen, 2010). These social constructs including: *self-esteem* (Entwistle and Ramsden, 1983), and intrinsic *motivation* (Dweck, 1986; Lau and Roeser, 2008) have been argued to affect individuals’ ability and confidence to learn new information. While research has sought to explore the role of individual differences in learning ability, it has largely overlooked the role of these social constructs on learning ability across the lifespan (cf. Hutchens et al., 2013). The individual difference measures consisted of the following items: Deep vs. surface learning style, strategic approach, achievement motivation, and self-esteem.

To test the ecological validity of individual difference questionnaires, we conducted pilot trials with 60 (30 young and 30 older) participants. Participants were asked to rate the questions included in the individual difference measures for clarity. Items rated highly by most participants as clear and comprehensible were included in the questionnaires used. These measures were then administered to the 60 participants in our study. In order for the individual differences measures to be consistent with the scoring of the cognitive variables, three of the scales (strategic approach, AMT, and self-esteem) were re-coded; i.e., a low score indicates a high rating on the scale. The individual difference measures consisted of the following scales:

#### Deep-Surface Learning Style

Participants were presented with five statements, taken from Tait et al.’s (1998) Approaches and Study Skills Inventory for Students (ASSIST; Tait et al.’s, 1998). These included, “I look at evidence carefully to reach my own conclusions;” and “What I have learned frequently seems unrelated to other bits and pieces” (reverse coded). All items were scored 1 = *disagree completely* through to 5 = *agree completely*. The mean of the 5-items was taken as a measure of learning style with high scores indicating deep learning style, while low scores indicating a “non-deep” surface learning style (∀ = 0.78).

#### Strategic Approach

The strategic approach scale comprised three subscales, also adapted from the ASSIST scale (Tait et al.’s, 1998). These subscales consisted of: (1)* a strategic approach scale*, with items such as: “I pay careful attention to any advice I am given, and try to improve my understanding”; (2) *an effort scale*, with items including, “I generally keep working hard even when things aren’t going all that well”; and (3) *an organized scale*, with items like, “I carefully prioritize my time to make sure I can fit everything in.” All items were scored 1 = *disagree completely* through to 5 = *agree completely*. After recoding, the mean of all of the items across the three subscales was taken as a measure of a “strategic learning approach”, with low scores indicating a strategic learning approach towards learning (∀ = 0.75).

#### Achievement Motivation

The achievement motivation scale consisted of 6-items, such as: “I work to cultivate people who will be helpful to me in the future.” All items were scored 1 = *disagree completely* through to 5 = *agree completely*. After recoding, the mean of the items was taken as a measure of achievement motivation, with low scores indicating high achievement motivation (∀ = 0.69).

#### Self-Esteem

Self-esteem was measured using a 10-item scale (Rosenberg, 1965) including items like, “On the whole, I am satisfied with myself.” All items were scored 1 = *disagree completely* through to 5 = *agree completely*. After recoding, the mean of the items was taken as a measure of self-esteem, with low scores indicating positive self esteem (∀ = 0.78).

### Cognitive Tasks

We used a battery of cognitive tasks to measure a range of abilities that have been suggested to affect learning ability. In particular, previous work provides evidence for the importance of working memory (WM) capacity (Law, 2000), attention span (Hambrick and Engle, 2002), processing speed (Salthouse and Ferrer-Caja, 2003; Mayes et al., 2009) and cognitive inhibition (St Clair-Thompson and Gathercole, 2006) in learning ability in young adults. Further, work in older adults suggests that individual differences in cognitive decline may drive age related differences in perceptual learning ability (Hultsch et al., 1990). Here, we tested the young and older participants on the following tasks: WM, cognitive inhibition, selective and divided attention (DA) and multiple object tracking.

#### Memory: Working Memory Task

The WM task was designed based on the sequential WM task used by Luck and Vogel (Luck and Vogel, 1997). Colored dots were displayed on a gray background for 500 ms, followed by a 1000 ms delay. After the delay, the dot display re-appeared with one of the dots highlighted by a white square. Participants reported whether the highlighted dot had remained the same color on the second presentation. An initial display of two dots was used. By using a two down one up staircase and a step size of 1 we manipulated the number of dots in the display, resulting in 70.7% performance. For example; each time the participant had two responses correct in a row an additional dot would be added to the next trial’s display, while for every incorrect answer, one dot was removed from the display for the next trial. WM thresholds (i.e., number of dots in the display) were calculated by averaging the last two-third reversals in each staircase. For each trial, each dot was randomly assigned a color, and one dot was randomly chosen as the target. Each dot had a radius of 12 pixels and dots were displayed in random locations within a 10 × 10 grid (jittered +/− 10 pixels). Each run consisted of 10 staircase reversals, participants completed 3 runs, after which we computed the average threshold as their WM score. In this task, a higher score (greater number of items in display) denotes better performance.

#### Inhibition: Stop-It Task

We used the Stop-It task developed by Verbruggen et al. (Verbruggen et al., 2008), which measures response inhibition based on the stop-signal paradigm (Lappin and Eriksen, 1966). Participants were asked to respond to the “go signal” (a white square or a circle presented in the center of a black background, displayed for 250 ms) by pressing a right or left response key to indicate the shape’s identity. The “go signal” remained on the screen until the participant responded, or for a maximum of 1,250 ms. “Go signals” were separated by a white fixation cross, displayed for 2,000 ms. On 25% of the trials a “stop signal” (750 Hz auditory tone, presented for 75 ms) was presented after the “go signal” had been displayed, instructing participants to inhibit their response for that trial. This delay (SSD: Stop Signal Delay) varied across trials. It was initially set at 250 ms and adjusted continuously using a staircase tracking procedure: When inhibition was successful, SSD increased by 50 ms; when inhibition was unsuccessful, SSD decreased by 50 ms. The task comprised of 3 blocks which consisted of 64 trials each. We used the latency of the stop process (SSRT), as initiated by the stop signal (see Verbruggen et al for full details), as our measure of cognitive inhibition. Poor inhibition is indicated by a slow SSRT; that is lower scores (faster SSRT) indicate good performance on this task.

#### Attention: Multiple Object Tracking Task

Multiple object tracking tasks measure human attention span and short term memory. We designed a task similar to that used by Sekuler et al. (2008). For each trial, a display of 10 stationary, blue and red dots (radius of 8 pixels each) appeared on a gray background for 1000 ms. After this initial fixation period the target red dots turned blue and all dots moved around the display for 5000 ms. Once the dots were stationary, a number (1–10) appeared in each blue dot. Participants were asked to indicate where the red dots were in the display by entering their corresponding numbers. This task consisted of 80 trials (20 per condition comprising 2, 3, 4 and 5 red target dots). Dots were assigned to random positions within a 10 × 10 grid (with a jitter of +/− 10 pixels), ensuring all dots were at least 16 pixels apart. Target locations and the angle at which each dot should move were randomly assigned for each trial. Dots had a maximum velocity of 2 pixels per frame and the Euclidean distance between two disks was always less than the diameter of a disk. If dots collided in the display, motion direction was altered. Task performance was measured by plotting the percentage of correctly tracked dots for each condition and calculating the slope of the fitted performance across conditions. Thus, a lower score (shallower slope) indicates better performance in this task.

#### Attention: Useful Field of View

Useful Field of View (Visual Awareness Inc.) is a task that assesses three attentional processes: processing speed, DA and selective attention (SA). This version of the task is explained in full by Edwards et al. (2005, 2006), who also report a test-retest reliability of 0.74. Each trial consisted of 4 stages: (1) a fixation bounding box (1 s duration), (2) the test stimuli (variable duration; see below), (3) a white noise visual mask to control for after images (1 s duration), and (4) the response screen (displayed until a response is made). Participants responded using the mouse. The first test, “processing speed”, required participants to identify a centrally presented stimulus. This stimulus (a silhouette of a 2 cm × 1.5 cm of a car or a truck) was presented on a black background inside a 3 cm × 3 cm white bounding box. Participants were asked to indicate whether the central stimulus comprised a car or truck by mouse click. The second task, “divided attention”, required participants to identify the central stimulus (car vs. truck), and also identify the location of a simultaneously presented peripheral stimulus (2 cm × 1.5 cm silhouette of a car). This peripheral stimulus was fixed at 11 cm from the central stimulus at one of 8 radial locations. The third task “selective attention” followed the same procedure as “divided attention” but the target stimuli were presented in the context of distractors (47 triangles of the same size and luminance as the targets). Participants were instructed to ignore the triangles, and indicate whether the central stimulus comprised a car or a truck, as we all the location of the peripheral target. Using a double staircase method the duration of the display within each task varied between 16.7 ms and 500 ms. This allowed us to establish the minimal display duration at which the participant could correctly perform each of the three tests 75% of the time. This means that a lower score (shorter duration) indicates better performance. Further, this manipulation allowed for the tasks to be adjusted for difficulty across age groups appropriately.

### Data Analysis: Partial Least Squares Regression Modeling

PLS regression is a component based multivariate statistical technique that allows predicting single or multiple response variables Y (i.e., threshold reduction) from highly correlated or collinear multiple explanatory variables X (i.e., cognitive abilities and individual differences variables, respectively; Wold, 1985). In contrast to principal components regression, the goal of PLS regression is not to form only components that capture most of the information in X, but components that are also predictive of Y. As such, the algorithm reduces the dimensions of X through a weighted linear combination of X variables to form orthogonal components that are correlated to the dependent variable. The analysis shows how much of the variation in Y and is accounted for by each additional component obtained from X. For Y (threshold reduction) cumulative variance can be interpreted in the same way as unadjusted R-square. Adjusted R-square shows the adjusted version of the cumulative Y variance.

## Results

To provide a sensitive and controlled measure of perceptual learning, we asked young and older participants to discriminate global visual forms (radial vs. concentric) defined by simple patterns of dots (Glass patterns). We manipulated participants’ ability to perceive these global patterns by varying: (a) the amount of background noise (i.e., randomly placed dots), and (b) the similarity between global forms, using linear morphing between concentric and radial patterns. To quantify the effect of learning, we used the following index:

$$\text{Threshold reduction = }\frac{Th_{pre}-Th_{post}}{Th_{pre}-Th_{post}}$$

where Th(session) is the mean shape discrimination threshold for each session.

**Figure 2:**
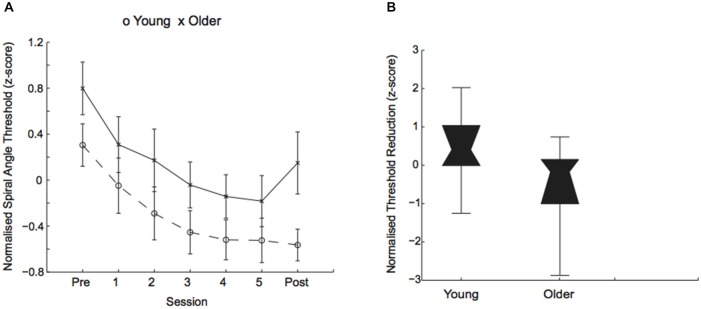
**Behavioral improvement in visual discrimination task. (A)** Normalized (*z*-score) thresholds (deg of spiral angle at 79.4% threshold performance) across training sessions for young (circles) and older (crosses) participants. **(B)** Normalized (*z*-score) threshold reduction (i.e., difference in thresholds between post- and pre-training) for young and older participants. Box plots show individual variability in learning performance: in threshold performance (*z*-scores) ranged from −1.25 to 2.02 for younger adults and from −2.87 to 0.74 for older adults. The upper and lower error bars display the minimum and maximum data values and the central boxes (“bowties”) represent the interquartile range (25th to 75th percentiles). The notch of the “bowtie” represents the median.

Performance on this form discrimination task improved through training in both young and older adults (i.e., a reduction in the signal needed for 79.4% threshold performance was similar across age groups) with overall better performance for young than older participants (Figure 2A). In particular, a mixed design ANOVA, showed a significant main effect of session (*F*_(1,58)_ = 147.82, *p* = 0.001) and age (*F*_(1,58)_ = 14.84, *p* = 0.002), but no significant interaction between age and session (*F*_(1,58)_ = 2.62, *p* = 0.11). Interestingly, we observed strong individual variability in performance for both young and older adults (Figure 2B).

### Individual Differences

To investigate the sources of this individual variability in learning improvement, we used the battery of cognitive tests (multiple object tracking: MOT, DA, SA, WM, and cognitive inhibition) and theory-grounded individual differences measures (learning style: deep vs. surface, strategic approach, self-esteem, and achievement motivation). Consistent with previous studies (Hedden and Gabrieli, 2004), these measurements showed that older adults differ in cognitive abilities and social profile from young adults (Table 1). Specifically, older adults had significantly lower performance in: cognitive inhibition (*t*_(58)_ = −2.454, *p* = 0.019), divided attention (*t*_(49)_ = −3.242, *p* = 0.003), selective attention (*t*_(48)_ = −6.288, *p* = <0.001) and WM (*t*_(58)_ = 4.046, *p* = <0.001) tasks compared to young adults. Further, older adults were more likely to engage in deep learning (*t*_(54)_ = −2.715, *p* = 0.009), and rely on achievement motivation as a drive for learning (*t*_(55)_ = −4.291, *p* = <0.001) than young adults (See Table 1).

**Table 1:** **Performance in cognitive and individual differences measures**.

	Age group	Mean	Std. error
Cognitive inhibition*	Young	280.55	5.84
	Older	315.81	13.13
Multiple object tracking	Young	−23.10	0.86
	Older	−20.08	1.28
Divided attention**	Young	33.48	6.31
	Older	85.77	14.84
Selective attention***	Young	87.05	13.18
	Older	212.67	14.62
Working memory***	Young	4.56	0.20
	Older	3.38	0.21
Learning style*	Young	3.59	0.10
	Older	4.00	0.10
Strategic approach	Young	3.94	0.06
	Older	3.83	0.11
Achievement motivation***	Young	3.21	0.12
	Older	3.87	0.09
Self esteem	Young	0.02	0.14
	Older	3.87	0.08

Mean scores and standard errors for each cognitive and individual difference measures for young and older participants. All variables, other than working memory, are coded so that lower scores indicate higher performance on cognitive tasks and a higher rating on individual differences measures. Working memory is coded so that higher scores indicate high performance. The learning style (surface/deep) variable is coded so that a low score (1) indicates surface learning while a high score (5) indicates deep learning. Asterisks indicate a significant difference between the two age groups. *p < 0.05, **p < 0.01, ***p < 0.001.

**Figure 3:**
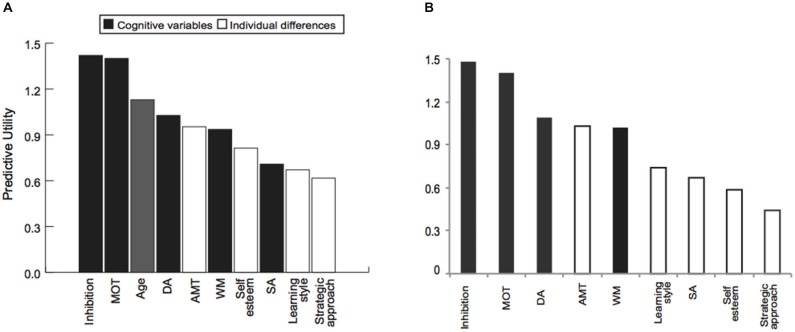
**The role of cognitive and social profiles in learning variability**. Outcome of the PLS model showing predictive utility of each variable in the PLS model when **(A)** age is included or **(B)** excluded from the model. Predictive utility values indicate the relative importance of each variable in predicting learning performance (i.e., threshold reduction). Variables included in the model comprise: (i) cognitive abilities measures (black bars): cognitive inhibition, multiple object tracking ability (MOT), Age, divided attention (DA), selective attention (SA), working memory (WM); (ii) individual differences measures (white bars): achievement motivation (AMT), self-esteem, learning style and strategic approach.

We then sought to relate the measured cognitive and social abilities to individual learning ability across age groups, using a partial least squares (PLS) regression model. This procedure allows us to test the predictive utility of a model even in smaller datasets (30 young vs. 30 older adults), that otherwise could be subjected only to correlational analysis. Our results show that a combination of cognitive abilities (i.e., performance in cognitive inhibition, MOT, DA, WM tasks), and individual differences variables (i.e., extent to which one engages in deep vs. surface learning, motivational impetus, and higher self-esteem), account for 60% of the variance in performance threshold reduction independent of age (Table 2). Excluding age from the PLS model showed similar results; that is, cognitive and social variables alone accounted for a significant portion (i.e., 58%) of the explained variance in performance. Further, we found that learning is best predicted by the ability to inhibit irrelevant information (cognitive inhibition), and select targets (MOT). That is, PLS values are higher for these cognitive variables when age is included or excluded from the model (Figure 3). Removing these cognitive variables from the model reduces substantially the explained variance (i.e., 46%).

**Table 2:** **Variance predicted by the PLS model**.

Latent factors	Adjusted R-square
Latent factor 1	0.547
Latent factor 2	0.599
Latent factor 3	0.600
	Latent factors		
	Latent factor 1	Latent factor 2	Latent factor 3
Cognitive inhibition	−0.381	−0.297	−0.162
MOT	−0.330	−0.474	−0.311
Divided attention	−0.392	0.259	0.236
Selective attention	−0.249	0.483	−0.663
Working memory	0.351	−0.223	0.035
AMT	−0.351	0.129	0.517
Self esteem	−0.292	0.045	0.358
Learning style	−0.217	−0.068	0.226
Age	−0.429	0.325	−0.248
Strategic approach	−0.051	−0.545	0.395

*Adjusted R^2^ values, and loadings of each of the cognitive and individual differences measures onto each of the components in the PLS model*.

### Comparing Strong and Weaker Learners

**Figure 4:**
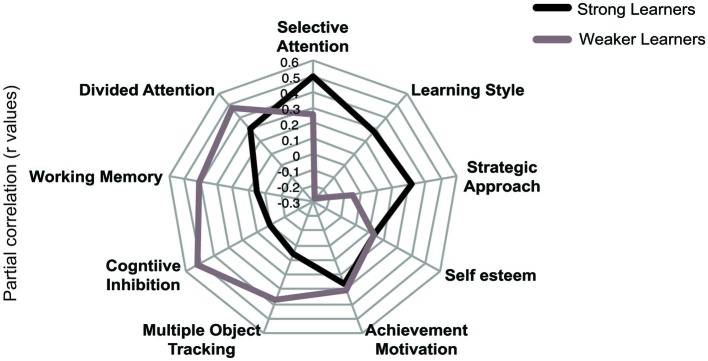
**Comparing profiles for strong and weaker learners**. Results of partial correlations (*r* values) for “strong” and “weaker” learners. Threshold reduction is correlated with cognitive and individual differences measures, while controlling for pre-training performance in the visual discrimination task and age. Note that for graphical representation purposes, the signs of any negatively coded variables have been reversed, indicating that increased scores in cognitive and individual difference measures correlate with increase in threshold reduction.

To provide an alternative approach to the study of individual variability, we compared strong and weaker learners independent of age using partial correlations. To assign participants to these performance groups we first normalized threshold reduction scores within each age group. Participants with a normalized score (z-score) of above 0 were classified as strong learners while those with a score below 0 were classified as weaker learners. We correlated cognitive variables and individual difference measures with threshold reduction, while controlling for pre-training performance in the visual form discrimination task and age. This analysis revealed that different cognitive abilities predict individual learning ability in strong vs. weaker learners (Figure 4), despite similar performance in these measures between groups (Table 3). Learning improvement (i.e., higher threshold reduction) correlated with higher SA scores (*r*_(24)_ = −0.499, *p* = 0.011) for strong learners, while with cognitive inhibition (*r*_(27)_ = −0.522, *p* = 0.006), working memory (*r*_(30)_ = 0.411, *p* = 0.037) and divided attention (*r*_(24)_ = −0.479, *p* = 0.021) scores for weaker learners. These results were supported by power calculations indicating that we have 80%–87% power to detect correlations of 0.48 for the sample sizes included in this study.

**Table 3:** **Cognitive and individual differences measures for strong and weaker learners**.

			Correlations with threshold reduction (*r* values)	
	Mean	*n*-size (strong in brackets)	Strong learners	Weaker learners
Cognitive inhibition	294.32 (302.60)	27 (30)	−0.003	−0.522**
Multiple object tracking	−22.28 (−20.80)	27 (30)	−0.056	−0.376
Divided attention	56.33 (68.78)	24 (26)	−0.306	−0.479*
Selective attention	160.07 (148.81)	24 (26)	−0.499*	−0.251
Working memory	3.97 (3.97)	27 (30)	0.054	0.411*
Learning style	3.74 (3.84)	27 (30)	−0.281	0.279
Strategic approach	3.79 (4.01)	27 (30)	−0.316	0.052

*Mean performance and standard errors for cognitive variables and individual difference measures. Results for strong and weaker learners are displayed outside vs. inside brackets, respectively. Partial correlations of cognitive and social variable scores with threshold reduction are reported separately for strong and weaker learners. All variables with the exception of working memory are coded so that a low score indicates strong performance; that is, negative correlations signal that high scores/ratings on these variables correlate with increased threshold reduction. For working memory, high performance correlates with increased threshold reduction, as shown by the positive correlation. For learning style, a negative correlation indicates that surface style correlates with better threshold reduction, while a positive correlation indicates that deep learning style correlates with better threshold reduction*.

### Modulatory Effects of Age

Our findings so far suggest that both cognitive and individual difference variables play a role in determining learning ability across age. However, this does not rule out the possibility that age may modulate the relationship between cognitive and individual difference variables. To test this hypothesis, we conducted additional analysis using threshold reduction as dependent variables in the PLS model. Our results showed that age modulates the importance of cognitive (i.e., cognitive inhibition, attention span as assessed by divided and SA) and social (i.e., learning style, strategic approach, motivational impetus and self-esteem) variables in predicting learning ability (Table 4), suggesting that some variables become more important in predicting threshold reduction at older age. Specifically, the pattern emerging is that age directly impacts cognitive inhibition, learning style and esteem. Further, older adults drew on a different style to guide their learning of novel information as compared to that of younger participants: they are motivated to look for more detail and a greater overall understanding of the task. Additionally, higher personal self-esteem is significantly more important for higher learning improvement in older participants. That is, older adults rely more on deep or strategic learning strategies than young adults, as self-esteem mostly and to a lesser extent cognitive abilities decrease with age.

**Table 4:** **(A)** Adjusted R-square values for PLS Regression without Age as an independent measure. **(B)** PLS model with threshold reduction as dependent variable and age as a moderator.

(A)
Latent factors	Adjusted R-Square
1	0.421
2	0.564
3	0.588
**(B)**	**Dependent variables**	
	**Unstandardized estimates**	
**Independent variables**	**Threshold reduction**	**Age (moderator of independent variable)**
Cognitive inhibition	0.868	1.111**
MOT	1.248	0.666
Divided attention	0.962	1.030*
Selective attention	0.941	1.056*
Working memory	1.209	0.736
Self-esteem	0.438	1.345***
Learning style	−0.720	−1.241***
Strategic approach	1.000	1.134*
AMT	0.9380	1.059*

Unstandardized estimates for cognitive and individual difference measures (left column) and the interaction between these independent variables and age (right column). Increased values (from left to right column) indicate the moderating effect of age on the independent variables. For learning style, a low score indicates surface learning and a high score indicates deep learning; thus a negative sign indicates that strong “surface learning style” predicts strong learning performance. Note: *p < 0.05, **p < 0.01 ***p < 0.001.

## Discussion

Our findings provide evidence that an individual’s cognitive and social skills profile rather than age *per se* predicts the ability to improve in perceptual judgments through training. Testing for age related differences alone may obscure the role of these variables in measuring individuals’ learning ability. In contrast to previous studies focusing on age differences (Ball and Sekuler, 1986; Richards et al., 2006; Andersen et al., 2010; Bower and Andersen, 2012), we demonstrate that attentional capacity, learning style and intrinsic motivation are critical for improving in perceptual tasks through training. Interestingly, our results show that strong learners are better able to select the most appropriate cognitive strategy (i.e., SA to targets) to improve at the task in hand (i.e., visual form discrimination in noise), while weaker learners rely on more general cognitive strategies.

Our work focuses on learning as a result of training on perceptual tasks. Previous studies have suggested that aging may result in reduced efficiency (Bennett et al., 1999), increased internal noise (Bennett et al., 2007) or reduced tolerance to external noise (Bower and Andersen, 2012) affecting performance in perceptual tasks. However, learning in young adults has been suggested to enhance performance efficiency (Gold et al., 2004), improve exclusion of external noise and reduce internal noise (Dosher and Lu, 1999). Extending beyond these previous studies, we show that perceptual learning is influenced by executive functions (i.e., the ability to inhibit distractors and select task-relevant features) and social attitudes (i.e., strategic or deep learning). These findings are consistent with previous studies (Kuai and Kourtzi, 2013) showing that visual form learning in aging is limited by visual selection processes rather than fine feature processing. Further, the ability to suppress irrelevant background information has been shown to deteriorate with age (Betts et al., 2005, 2009) possibly due to weakening of inhibitory processes (Leventhal et al., 2003; Hua et al., 2010) or attentional functions in aging (Ball et al., 1990; Kane et al., 1994). Interestingly, in our previous work (Mayhew and Kourtzi, 2013) we have shown that visual shape learning engages primarily parietal regions in older adults, suggesting a stronger role of attentionally-guided learning that enhances the perceptual salience of behaviorally relevant targets in cluttered scenes (Gottlieb et al., 1998; Corbetta and Shulman, 2002; Roelfsema and van Ooyen, 2005; Mevorach et al., 2010).

Our findings have potential implications for understanding compensatory brain mechanisms that may support individual ability for learning in older age. Understanding the socio-cognitive profile of individuals and how it influences learning ability is critical for determining the brain mechanisms that underlie individual variability and may support better learning in some older adults than others. For example, our findings suggest that older participants find it more difficult to inhibit irrelevant details. It is possible that older adults may attempt to compensate for this change in cognitive capacity by drawing on strategic learning, or the use of deep learning strategies focusing on more thorough understanding of new information. In future work, it would be interesting to test whether varying the training task recruits different socio-cognitive variables as best predictors of learning ability. It is possible that cognitive inhibition and attention are critical when detecting targets from noise and discriminating highly similar stimuli. However, other cognitive variables (e.g., WM) may be more important in the context of associative or probabilistic learning tasks. Further, learning in other domains, such as verbal or motor learning, may be influenced by a different set of socio-cognitive abilities. It may also be interesting to enrich the individual difference measures using a measure of control beliefs (Hutchens et al., 2013), as control beliefs may offer further insights into the factors that learners perceive as beneficial for their general learning ability.

Overall, our findings have practical relevance for the optimization of training programs targeting cognitive abilities and social attitudes, which are critical for improvement in perceptual tasks but more importantly for generalizing learning to real-life situations. Future research would investigate why older people may adopt different strategies to maximize learning; and how readily they may adopt alternate strategies for learning, if they diverge from the ones that they may feel comfortable employing.

## Conflict of Interest Statement

The authors declare that the research was conducted in the absence of any commercial or financial relationships that could be construed as a potential conflict of interest.
